# Supplementary material for: Clinical outcomes following shock team implementation for cardiogenic shock: a systematic review
Source: Egypt Heart J. 2024 Dec 30;76:163. doi: 10.1186/s43044-024-00594-z (PMC11685370; doi:10.1186/s43044-024-00594-z)
Supplement: Supplementary file 1 — Additional file 1 [file 43044_2024_594_MOESM1_ESM.docx]

**Clinical Outcomes Following Shock Team Implementation for Cardiogenic Shock: A Systematic Review.**

**Running Title.**

Efficacy of Cardiogenic Shock Team

**Authors.**

Mohamed Abdelnabi ^1,$^, Ahmed Saad Elsaeidy (Co-First author) ^2,$,*^, Aya Moustafa Aboutaleb ^3^, Amit Johanis ^4^, Ahmed K. Ghanem ^5^, Hazem Rezq ^6^, Basel Abdelazeem ^7^

**Affiliations.**

1. Department of Clinical Pharmacy, University of Michigan, Ann Arbor, Michigan, USA.
2. Faculty of Medicine, Benha University, Benha, Egypt.

https://orcid.org/0000-0002-1643-9750

1. Faculty of Medicine, Zagazig University, Zagazig, Egypt.
2. School of Medicine, Creighton University, Phoenix, USA.
3. Cardiology Department, the Lundquist Institute at Harbor-UCLA Medical Center, Torrance, CA, USA.
4. Faculty of Medicine, Al-Azhar University, Cairo, Egypt.
5. Department of Cardiology, West Virginia University, Morgantown, WV, USA, 26505.

**$ Equal contributions**

*** Corresponding author:**

Ahmed Saad Elsaeidy

Faculty of Medicine, Benha University, Benha, Egypt.

Tel: 00201555865546

AhmedSaadElsaeidy@gmail.com

<https://orcid.org/0000-0002-1643-9750>

**Appendix: Search strategy for each database.**

| Database | Search Terms | Search Field | Results number |
| --- | --- | --- | --- |
| PubMed | #1 ((Hospital Medical Emergency Team) OR (Rapid Response Team) OR (Cardiac Crash Team) OR (Cardiogenic shock team) OR (Code Team) OR (Shock team) OR (Patient Care Team) OR (Medical Care Team) OR (Healthcare Team) OR (Health Care Team) OR (Interdisciplinary Health Team) OR (Multidisciplinary Health Team) OR (Multidisciplinary Care Team) OR (Extracorporeal Membrane Oxygenation team)) OR (ECMO team)  #2 (Cardiac shock) OR (Cardiogenic Shock)  #3 (#1) AND (#2) | All Field | 768 |
| Web of Science Core Collection | #1 ((((((((((((((ALL=(Hospital Medical Emergency Team)) OR ALL=(Rapid Response Team)) OR ALL=(Cardiac Crash Team)) OR ALL=(Cardiogenic shock team)) OR ALL=(Code Team)) OR ALL=(Shock team)) OR ALL=(Patient Care Team)) OR ALL=(Medical Care Team)) OR ALL=(Healthcare Team)) OR ALL=(Health Care Team)) OR ALL=(Interdisciplinary Health Team)) OR ALL=(Multidisciplinary Health Team)) OR ALL=(Multidisciplinary Care Team)) OR ALL=(Extracorporeal Membrane Oxygenation team)) OR ALL=(ECMO team)  #2 (ALL=(Cardiogenic Shock)) OR ALL=(Cardiac shock) #1 AND #2 | All Field | 779 |
| Scopus | ( TITLE-ABS-KEY ( ( ( ( ( ( ( ( ( ( ( ( ( ( ( hospital  AND medical  AND emergency  AND team )  OR  ( rapid  AND response  AND team ) )  OR  ( cardiac  AND crash  AND team ) )  OR  ( cardiogenic  AND shock  AND team ) )  OR  ( code  AND team ) )  OR  ( shock  AND team ) )  OR  ( patient  AND care  AND team ) )  OR  ( medical  AND care  AND team ) )  OR  ( healthcare  AND team ) )  OR  ( health  AND care  AND team ) )  OR  ( interdisciplinary  AND health  AND team ) )  OR  ( multidisciplinary  AND health  AND team ) )  OR  ( multidisciplinary  AND care  AND team ) )  OR  ( extracorporeal  AND membrane  AND oxygenation  AND team ) )  OR  ( ecmo  AND team ) ) )  AND  ( TITLE-ABS-KEY ( ( cardiac  AND shock )  OR  ( cardiogenic  AND shock ) ) ) | TITLE-ABS-KEY | 948 |
| Embase | #3.  #1 AND #2  #2.  'cardiac shock':ti,ab,kw OR 'cardiogenic       shock':ti,ab,kw  #1.  'hospital medical emergency team':ti,ab,kw OR       'rapid response team':ti,ab,kw OR 'cardiac crash       team':ti,ab,kw OR 'cardiogenic shock       team':ti,ab,kw OR 'code team':ti,ab,kw OR 'shock       team':ti,ab,kw OR 'patient care team':ti,ab,kw OR       'medical care team':ti,ab,kw OR 'healthcare       team':ti,ab,kw OR 'health care team':ti,ab,kw OR       'interdisciplinary health team':ti,ab,kw OR       'multidisciplinary health team':ti,ab,kw OR       'multidisciplinary care team':ti,ab,kw OR       'extracorporeal membrane oxygenation       team':ti,ab,kw OR 'ecmo team':ti,ab,kw | All Field | 129 |
| Cochrane | #1 (Hospital Medical Emergency Team):ti,ab,kw OR (Rapid Response Team):ti,ab,kw OR (Cardiac Crash Team):ti,ab,kw OR (Cardiogenic shock team):ti,ab,kw OR (Code Team):ti,ab,kw  #2 (Shock team):ti,ab,kw OR (Patient Care Team):ti,ab,kw OR (Medical Care Team):ti,ab,kw OR (Healthcare Team):ti,ab,kw OR (Health Care Team):ti,ab,kw  #3 (Interdisciplinary Health Team):ti,ab,kw OR (Multidisciplinary Health Team):ti,ab,kw OR (Multidisciplinary Care Team):ti,ab,kw OR (Extracorporeal Membrane Oxygenation team):ti,ab,kw OR (ECMO team):ti,ab,kw  #4 #1 OR #2 OR #3  #5 (Cardiac shock):ti,ab,kw OR (Cardiogenic Shock):ti,ab,kw  #6 #4 AND #5 | Title, Abstract and Keywords | 84 |
